# Whispering-Gallery Mode Micro-Ring Resonator Integrated with a Single-Core Fiber Tip for Refractive Index Sensing

**DOI:** 10.3390/s23239424

**Published:** 2023-11-26

**Authors:** Monika Halendy, Sławomir Ertman

**Affiliations:** Faculty of Physics, Warsaw University of Technology, 00-662 Warszawa, Poland

**Keywords:** sensor, refractive index, whispering-gallery mode (WGM), micro-ring resonator, optical fiber, two-photon polymerization (2PP)

## Abstract

A micro-ring resonator structure was fabricated via the two-photon polymerization technique directly on a single-mode fiber tip and tested for refractive index sensing application. The micro-ring structure was used to excite whispering-gallery modes, and observations of the changes in the resonance spectrum introduced by changes in the refractive index of the environment served as the sensing principle. The proposed structure has the advantages of a very simple design, allowing for measurements in reflection mode, relatively easy and fast fabrication and integration with a single tip of a standard single-mode fiber, which allowed for quick and convenient measurements in the optical setup. The performance of the structure was characterized, and the resonant spectrum giving high potential for refractive index sensing was measured. Future perspectives of the research are addressed.

## 1. Introduction

Resonators of circular geometry, such as ring, sphere, disc, etc., exhibit the remarkable capability to confine light for many roundtrips within their confines, during which the light can have numerous interactions with the ambient environment [1,2,3]. This capability makes them very sensitive sensors. The resonant modes arising in such resonators are commonly referred to as whispering-gallery modes (WGMs).

Even though WGM resonators are already a relatively mature concept, ongoing extensive research continues to explore new advancements in terms of fabrication, coupling methods [4] or application in various fields [5,6]. Of particular interest is research related to coating WGM resonators with nanomaterials to tailor and enhance their sensitivity for specific types of analytes [7,8]. Noble metals, such as silver (Ag) or gold (Au), are deposited on the surface of WGM resonators to construct opto-plasmonic WGM microcavities, which provide enhanced capabilities that neither pure silica WGM resonators nor pure plasmonic resonators can individually offer.

The sensing principle in the case of resonator-based sensors relies on observing changes in the resonance spectrum they produce, which are prompted by changes in environmental parameters. The resonant frequencies manifest themselves by Lorentzian dips observed in the measured spectrum [9]. When environmental parameters change, a shift in resonant wavelengths can be observed, which constitutes one of the most utilized sensing mechanisms. The desired properties of a resonance-based sensor include the following:

- High sensitivity, which refers to achieving a significant shift in the resonance spectrum per refractive index unit (RIU).
- High Q-factor—the higher it is, the sharper the dips in the resonant spectrum, enabling more precise measurements. The Q-factor can be expressed as a ratio of the resonant wavelength $\lambda_{res}$ to the linewidth of the dip corresponding to that wavelength δλ, as described in Equation (1).
  (1) $$Q=\frac{\lambda_{res}}{\delta \lambda }$$
- High extinction ratio, meaning the difference in power between dips and peaks in the resonant spectrum. For this research, it should preferably be not smaller than 8 dB. Lower values of the extinction ratio may pose challenges for optical receivers in practical applications to recognize high and low signal levels [10].
- Free spectral range (FSR) is another parameter of resonator sensors, representing the distance between adjacent dips in the spectrum. The FSR should not be excessively narrow because the narrower it is, the more limited the measurement range is—the starting position of the observed dip will be replaced sooner by the subsequent dip.

In general, the condition of light resonance is closely related to the geometry of the resonator. This relation is described by Equation (2), where m = 1, 2, …, *λ_m_*—the resonant wavelength, *n*—the effective refractive index, *L*—length of the resonator. *nL* defines the optical length of the resonator.

(2) $$m\lambda_{m}=nL$$

This means that the resonant wavelengths are those with an integer multiple that is equal to the optical length of the resonator. Then, the FSR can be expressed by Equation (3).

(3) $$FSR={\lambda_{m}-\lambda }_{m+1}=\frac{{\lambda_{m}\lambda }_{m+1}}{nL}$$

From an application point of view, changes in a resonant spectrum can be attributed to many factors, like changes in temperature [10], pressure [11], humidity [12], refractive index or the presence of specific biomolecules [13,14,15]. Refractive index (RI) sensors stand as one of the most popularly constructed and extensively documented sensor types. Among their fundamental applications is determining substance concentration within a given solution [16,17,18]. A particularly interesting proposition in this matter are droplet-based WGMs, where a droplet of the examined substance serves as an optical cavity itself, increasing the light interaction with the examined substance [19]. By measuring the RI of the environment, we can also determine its other parameters, like pressure or temperature, as they influence the refractive index. Furthermore, RI sensors lay a robust foundation for the development of biological and chemical sensors after modification of their surface, e.g., by coating with dedicated materials that respond to the presence of specific biomolecules [20,21,22].

When it comes to achieved sensitivity, typical values documented for WGM micro-resonators made of silica or polymer (for integration with optical fibers) are equal to around 100–200 nm/RIU [23,24,25]. Coupling with plasmonic nanoparticles is often proposed as a method to easily enhance their sensitivity (possibly even up to five times) [26]. Another simple and popular resonator structure for fiber-integrated sensing applications is the Fabry–Perot cavity, with which a sensitivity of ~1058 nm/RIU was achieved [27], though this type of structure generally exhibits lower Q-factors. Some of the highest reported RI sensitivities were achieved by sensors based on photonic crystal fibers (PCFs) combined with plasmon resonance (reaching up to ~5000 nm/RIU) [28,29] and in a Sagnac-type interferometer (~12,500 nm/RIU), where an optical fiber was twisted to create a fiber loop and where the changes in the resonant spectrum depended on changes in light polarization inside the structure [30].

One of the simplest structures proposed for RI sensing applications is a sphere on top of a fiber fabricated using a fiber-melting process [31]. However, the measurement setup requires aligning the structure with a prism to couple the WGMs. In this paper, the proposed fiber-integrated WGM resonator structure for RI sensing applications comprises a ring resonator and two waveguides attached along the ring’s tangential—it creates a smooth transition between the waveguides and the resonator ring; therefore, the proposed coupling method is the direct waveguide coupling, which eliminates the need for additional coupling tools. Another advantage of this coupling method is that it facilitates fabrication on a standard single-core fiber. Integration with a single-core fiber constitutes a challenge when it comes to applying evanescent wave coupling, which typically yields superior resonance performance but usually requires the resonator structure not to be in contact with the coupling waveguide. The evanescent wave coupling is usually realized by printing the structure on a planar substrate [32] or in a D-shaped segment of fiber [33] and precisely fixing it in a package with a coupling waveguide (usually a taper) in an optimal distance [24,34]. This solution limits the area of the sensor, which interacts with the environment, as a fraction of it has to be in contact with the substrate. The procedure of cleaning such structures is also less convenient than in the case of structures in the form of a sensor tip (as presented here), which can be immersed directly into analyte and simply immersed in isopropanol in between subsequent measurements. 

Another challenge is that these fibers provide only one channel for both input and output. To overcome this limitation, multi-core fibers have been used [35], but compared to them, standard single-core fibers have the advantages of easy availability and affordability. 

## 2. Proposed Structure and Fabrication Process

**Figure 1:**
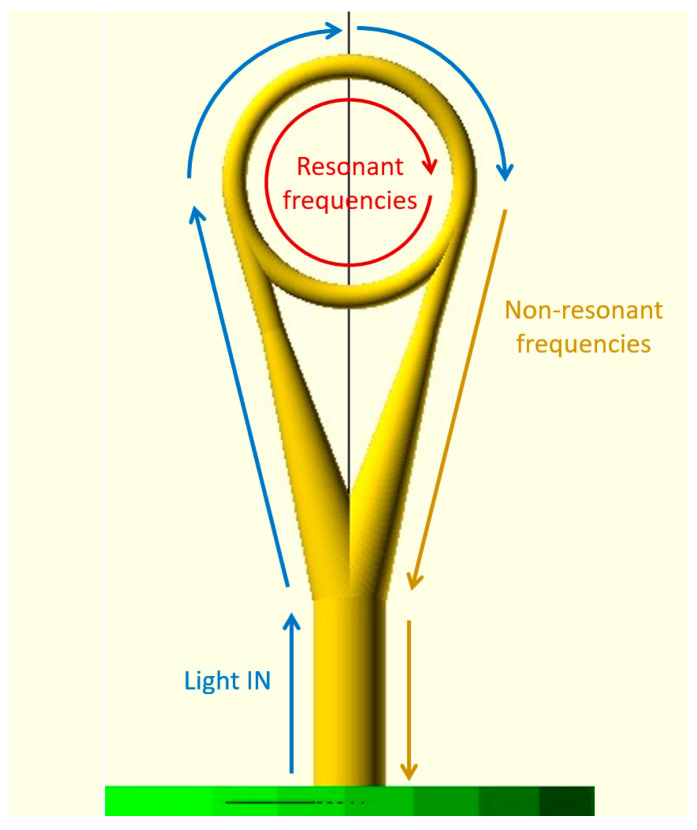
A schematic view of the proposed structure, illustrating light propagation inside it—scenario concerning only one direction. The red circular arrow indicates the “ring” path (mentioned later in the paper), and the yellow and blue arrows indicate the “loop” path.

A schematic view of the proposed structure is shown in Figure 1. The light propagating from the core, after coupling to the structure, is split into two arms (for clarity, the schematic view shows the situation for only one arm—only one direction of propagation is depicted), which then couple the light into the resonator ring along its tangential. The resonant frequencies of the injected spectrum will circulate inside the ring, and part of the light spectrum that does not fulfill the resonance conditions will couple back to the arms and propagate back to the core. The structure gradually tapers down, starting with a thickness of 9 μm (matching the core diameter), down to the thickness of 3 μm, corresponding to the thickness of the ring.

The structures were fabricated via the two-photon polymerization (2PP) technique, which is currently widely employed for nano- and microstructure fabrication [36,37,38]. The fabrication was realized with a commercial device, the Photonics Professional GT2 printer, provided by Nanoscribe GmbH & Co. (Karsluhe, Germany). KG. As a method for fabricating WGM resonators, 2PP can offer a very smooth surface for the structures, which is a crucial factor for effective light confinement, as it minimizes surface scattering.

The entire structure is remarkably simple and has a small volume, which makes the printing time very short (usually less than 2 min). Moreover, it requires no post-processing step other than the standard for 2PP printing development and rinsing, so the total fabrication time can be less than 20 min (for an experienced operator). 

**Figure 2:**
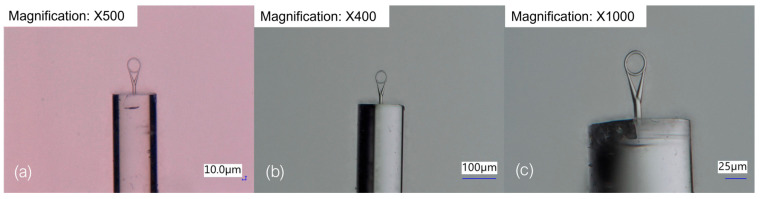
Microscope images of the printed structures with the ring diameter equal to (**a**) 40 μm, (**b**) 34 μm, (**c**) 28 μm.

Three structures with different ring diameters—40 μm, 34 μm and 28 μm—were printed. The resin used for fabrication was the IP-Dip2 resin supplied by Nanoscribe, which has a refractive index of 1.53 after polymerization. The printing was conducted using the objective with the highest available magnification, which was 63×. This resin-objective combination constitutes the so-called “Small Features Printing Set”. Digital microscope images of the printed structures are presented in Figure 2.

## 3. Experimental and Numerical Analysis

### 3.1. Characteristics of the Structure

**Figure 3:**
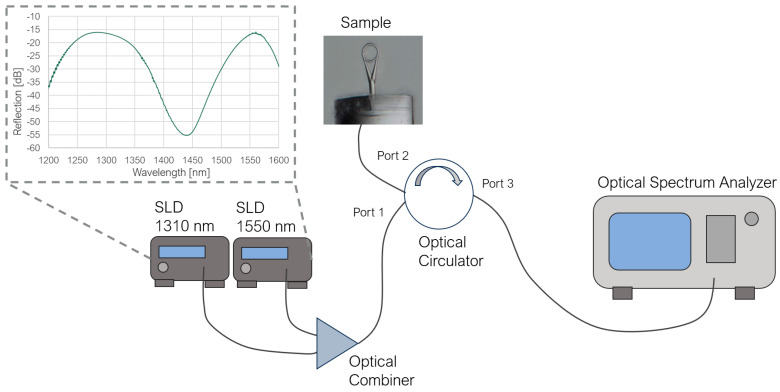
The measurement setup.

**Figure 4:**
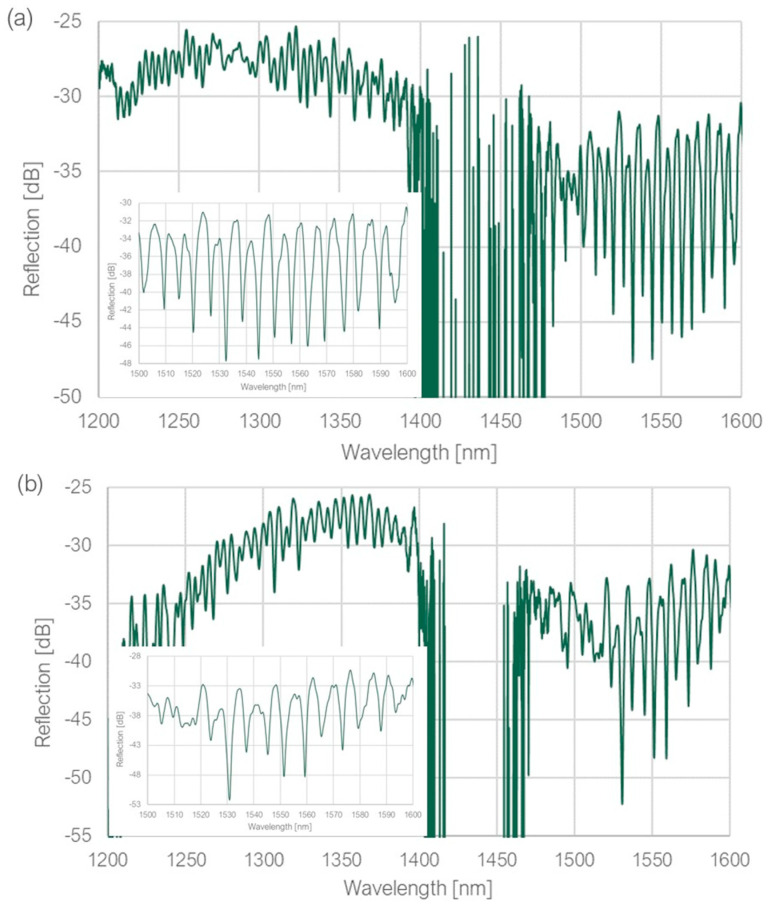

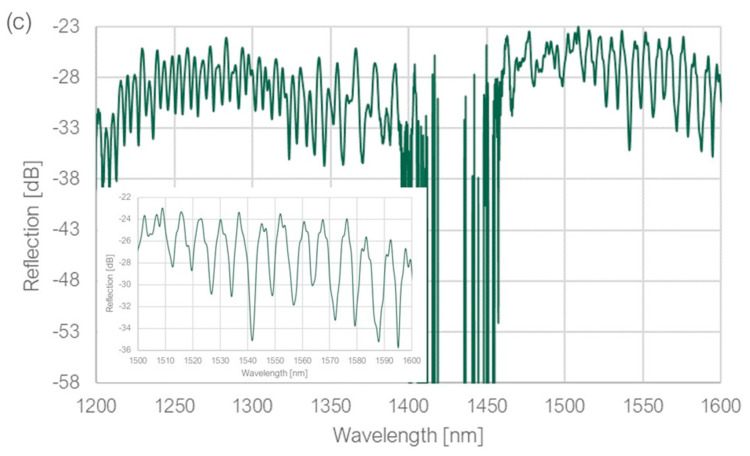
Measured spectra for the printed structures with the ring diameter equal to (**a**) 40 μm, (**b**) 34 μm, (**c**) 28 μm. Insets show the region of the best extinction ratio.

In the measurement setup (presented in Figure 3), the light was introduced by two SLD sources with central operating wavelengths of 1310 nm and 1550 nm. The source signals were transmitted to an optical combiner, where they were combined and transmitted to Port 1 of an optical circulator. The tested sample was connected to Port 2; the light after reflection traveled back to Port 2, then directly to Port 3 and to an Optical Spectrum Analyzer (Yokogawa AQ 6370C). All elements were connected using standard single-mode patch cords. The measured spectra across the whole wavelength range are shown in Figure 4; the attached insets provide a closer look at the region of the best extinction ratio. All spectra were measured with a resolution of 0.02 nm.

All measured structures were capable of exciting resonant modes with high extinction ratios, particularly in the wavelength range of 1500–1600 nm, achieving 16 dB, 18 dB and 11 dB for the structures of ring diameter equal to 40 μm, 34 μm and 28 μm, respectively. However, it was observed that the measured FSR values were not in agreement with the calculated theoretical values. Upon further investigation, it was determined that the observed dips might have originated from somewhere other than resonance inside the ring. The path of the uncoupled light (look at Figure 1), consisting of the coupling waveguide (the base and two tapered arms), together with part of the ring, formed a closed loop along which the light could resonate if the resonance condition from Equation (2) was satisfied. The calculated FSR values for both paths (along the ring and the “loop”) were compared to the measured values, as shown in Table 1.

Efforts were made to increase the amount of light coupled to the ring. Several numerical simulations were conducted to gain insight into the behavior of light inside the structure. The Ansys Lumerical FDTD solver was used to simulate the region of the waveguide-ring transition and the ring itself. The simulations were performed for the ring diameter of 16 μm—it was reduced to restrain the simulation time. The boundary conditions and the mesh settings of the simulation region were kept at their default values. The “Mode” source was used to inject guided modes into both waveguides.

**Figure 5:**
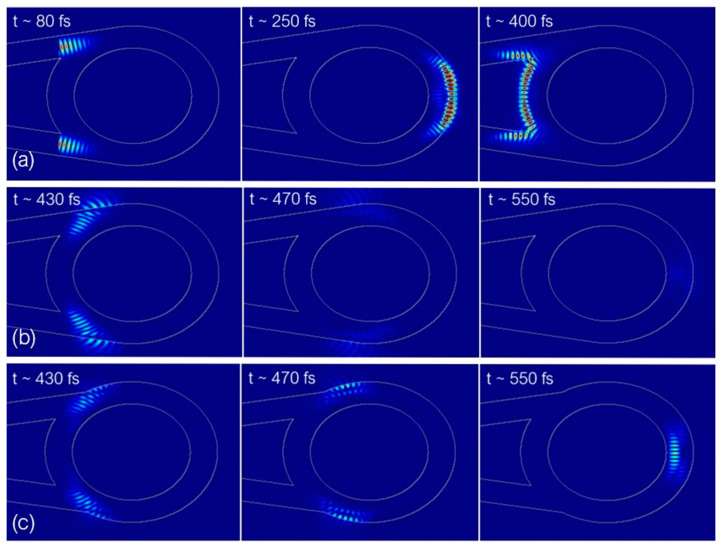
Lumerical “Movie” monitor screenshots of (**a**) light propagation during the first roundtrip, (**b**) light propagation during the second roundtrip, (**c**) light propagation during the second roundtrip after relocating waveguide arms.

Figure 5a illustrates the behavior of a beam with a spectral range around 1500–1600 nm during the first roundtrip in the ring, while Figure 5b illustrates what happens afterward. The beams, instead of continuing to propagate along the rim of the ring, encountered a flat surface where the angle of incidence no longer supported total internal reflection, resulting in light leakage. The proposed solution was to reposition the tapers so that they no longer created this flat segment on the sides of the ring. The tapers were moved closer together by a distance equal to 0.4 radians along the ring. The propagation during the second roundtrip after applying this modification is shown in Figure 5c.

**Figure 6:**
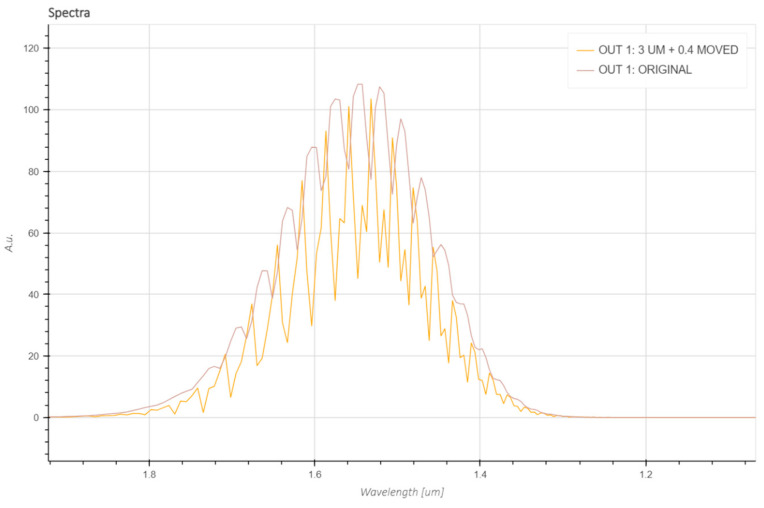
A comparison between the output resonance spectra provided by Lumerical “Time” monitor before and after aforementioned adjustment.

It can be observed that the intensity of the remaining light in the second roundtrip was significantly higher than previously, but for a more precise comparison, the fields were measured with a “Time” monitor, which provides information about the field in the wavelength function for a specific point or plane in the simulation region. Figure 6 illustrates a comparison between the obtained output fields.

The spectra obtained from simulations show that the extinction ratio increased after relocating the tapers, indicating that more light remained inside the ring in the form of WGMs. The FSR of the modes was equal to ~30 nm in the wavelength region around 1.55 μm. This FSR value is in agreement with the result obtained with Equation (3).

**Figure 7:**
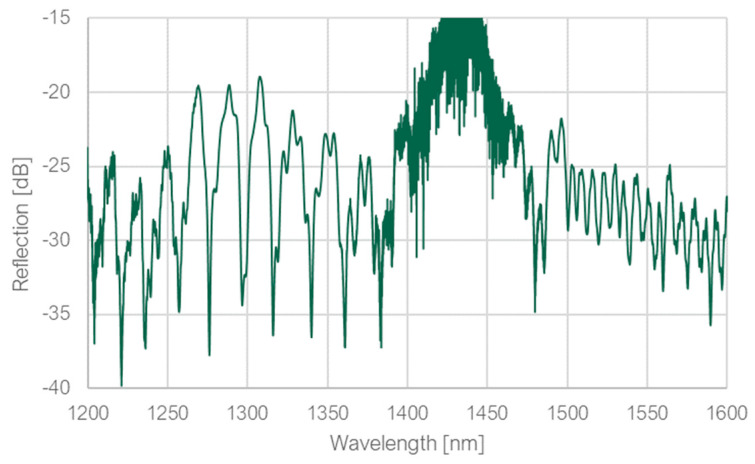
The measured spectrum of the simulated structure.

The simulated structure was printed to confirm if the simulation results could be replicated in reality. The ring diameter of 16 μm was maintained, but the distance between the ring and the fiber facet was slightly increased to lengthen the taper. It was necessary because the taper length decreases when the ring diameter is reduced (as the tangential is lowered). Maintaining a sufficiently long taper is crucial because if the taper is too short, power loss on it can be significant. The spectrum measured in the optical setup is shown in Figure 7.

Dips with a high extinction ratio of around 18 dB with a much wider FSR could be observed in the spectral range around 1310 nm. Therefore, several FSR values in this range were measured and compared to the theoretical values for both ring and loop to determine their source. The comparison is demonstrated in Table 2.

Based on the results presented in Table 2, it may be concluded that the deep dips in the wavelength range around 1310 nm were the result of resonance inside the ring. However, the resonance spectrum in this range was not uniform—narrower resonance modes could be observed inside the ones with FSR equal to ~20 nm. These modes can be the result of resonance along the loop. When it comes to the modes in the wavelength range around 1550 nm, it was difficult to distinguish whether they were the result of the resonance along the loop or the combination of both resonance spectra.

After these measurements, another adjustment to the structure was proposed with the intention of generating both ring and loop resonances. Having two resonance spectra intersecting each other is often desired for reference purposes, allowing the elimination of measurement deviations due to factors such as temperature. This procedure can be achieved with the use of two WGM resonators of different diameters [39] or by using two different resonator types [40]. 

**Figure 8:**
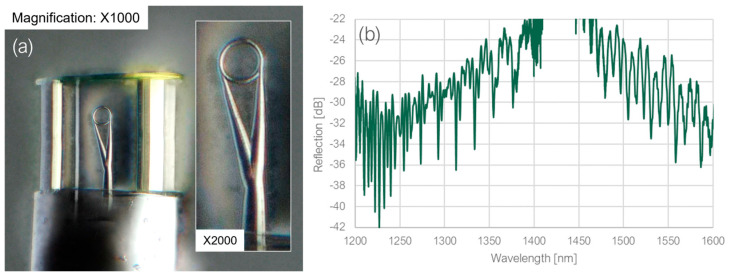
(**a**) A microscope image and (**b**) the measured spectrum for the thickened micro-resonator structure surrounded by protective enclosure.

The concept involved decreasing the thickness of the ring to 1 μm instead of relocating the tapers. By reducing the thickness, the incidence angle after a roundtrip, which previously caused the leakage, could still be altered in the desired way. At the same time, thanks to keeping the tapers at the tangential position of the ring, the loop resonance could potentially be observed again. The diameter of the ring remained at 16 μm. The printed structure is depicted in Figure 8, together with the measured spectrum. Due to the low thickness of the ring, the structure was printed together with a protective cavity.

In the wavelength range around 1310 nm, the ring dips were considerably deeper than the loop dips, whereas in the range around 1550 nm, they exhibited comparable depths. Nevertheless, both resonances could be clearly distinguished. The FSRs were once again measured and compared to the theoretical values, as presented in Table 3 and Table 4. The extinction ratio was much lower than in the previous structure, and the reason may be that the printed structure was slightly tilted.

### 3.2. Measurements with Varying Ambient Refractive Index

**Figure 9:**
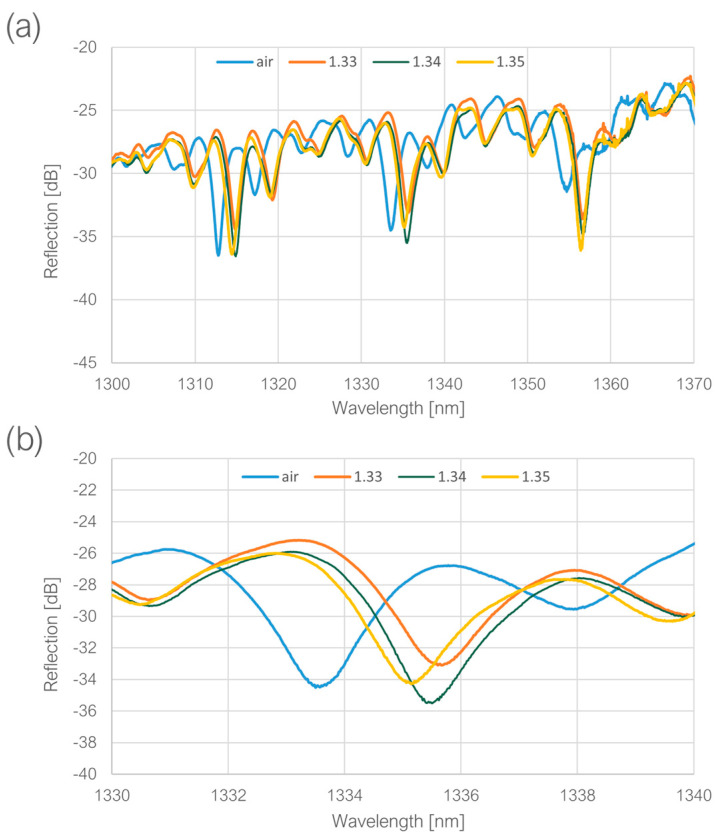
(**a**) Measured spectra for varying ambient RI, (**b**) shifts of a resonant dip for varying ambient RI.

For the RI measurements, a set of glycerin and water solutions of varying concentrations was prepared, resulting in RI (n_D_) values of 1.33, 1.34 and 1.35. The measured spectra are shown in Figure 9.

After immersion in the liquids, the extinction ratio increased slightly and reached around 8 dB, so the resonance dips could be easily discernible, as well as the resonance wavelength shift when the ambient RI was increasing. However, the sensitivity (S) in this RI range was calculated to be relatively low (according to Equation (4), where ${\Delta \lambda }_{res}$ represents the change in the resonant wavelength corresponding to the refractive index change Δn), standing at only 25 nm/RIU. Regarding the dip shift between the measurement in the air and in RI equal to 1.33, which amounted to 20.80 nm, the sensitivity within the RI range of 1–1.33 could be estimated at a somewhat more favorable rate of 63 nm/RIU. The Q-factor was measured to be above 1000.

(4) $$S=\frac{{\Delta \lambda }_{res}}{\Delta n}$$

## 4. Discussion and Conclusions

The proposed structure shows great potential for refractive index sensing. A resonance spectrum was successfully obtained with a quite high extinction ratio of the resonance dips (around 8 dB after immersion in RI ~ 1.33) and a quite high Q-factor (above 1000). Two intersecting resonance spectra constitute the output signal, which can be used for self-referencing and eliminating temperature influence on the measurements. The outstanding advantages of the proposed sensor, when compared to other propositions presented in the literature so far, are its simplicity, ease and short time of fabrication, and integration with a single tip of a single-core fiber. The measured sensitivity was rather low, which requires further improvements. The Lumerical software provides a possibility of simulating different RIs of the surrounding medium, so simulations of the sensor’s behavior after immersion can be performed in the future to search for geometrical adjustments, which would improve the sensitivity. One of the possible adjustments is simply increasing the ring diameter—longer distance of the sensing region means more interaction with the ambient medium.

Another particularly interesting proposition is coating the structure with noble metals to excite surface plasmon polaritons, inducing light enhancement and increasing its confinement at the structure surface. In general, in WGM resonators with no coating applied, a relatively small amount of light propagates outside of the resonator boundaries, and the usually achieved sensitivities are around 100–200 nm/RIU. Several studies have shown that coating with noble metals can increase the sensitivity of such structures significantly [23], but this procedure introduces a significant problem in structures coupled via evanescent wave coupling—the thicker the layer of the material, the larger the attenuation of the field during coupling, and the less energy that enters the resonator. The structure proposed in this research would have a particular advantage in this matter, as it utilizes direct waveguide coupling.

## Figures and Tables

**Table 1:** A comparison between measured and calculated FSRs for the structures with three different ring diameters.

Ring ϕ ^1^ [μm]	Ring OL ^2^ [μm]	Loop OL [μm]	λ_m+1_ and λ_m_ [nm]	Measured FSR [nm]	Calculated Ring FSR [nm]	Calculated Loop FSR [nm]
40.00	192.20	229.80	1550.40	6.40	11.78	7.00
			1556.80			
34.00	163.30	212.80	1551.40	7.80	13.61	7.58
			1559.20			
28.00	134.50	195.90	1548.80	8.00	16.19	8.21
			1556.80			

^1^ ϕ—Diameter. ^2^ OL—Optical length.

**Table 2:** A comparison between measured and calculated FSRs for the simulated structure.

Ring ϕ [μm]	Ring OL [μm]	Loop OL [μm]	λ_m+1_ and λ_m_ [nm]	Measured FSR [nm]	Calculated Ring FSR [nm]	Calculated Loop FSR [nm]
16.00	76.90	172.20	1276.12	20.74	21.53	6.40
			1296.86			
			1296.86	19.24	22.20	6.60
			1316.10			
			1316.10	23.80	22.94	6.82
			1339.90			
			1339.90	21.02	23.72	7.05
			1360.92			

**Table 3:** A comparison between measured and calculated ring FSRs for the thickened structure.

Ring ϕ [μm]	Ring OL [μm]	Loop OL [μm]	λ_m+1_ and λ_m_ [nm]	Measured FSR [nm]	Calculated Ring FSR [nm]
16.00	76.90	172.50	1312.70	20.92	22.77
			1333.62		
			1333.62	21.12	23.50
			1354.74		
			1354.74	21.76	24.26
			1376.50		
			1479.58	25.44	28.97
			1505.02		
			1505.02	26.02	29.98
			1531.04		
			1531.04	27.56	31.04
			1558.60		

**Table 4:** A comparison between measured and calculated loop FSRs for the thickened structure.

Ring ϕ [μm]	Ring OL [μm]	Loop OL [μm]	λ_m+1_ and λ_m_ [nm]	Measured FSR [nm]	Calculated Loop FSR [nm]
16.00	76.90	172.50	1364.36	6.1	7.22
			1370.46		
			1514.70	8.16	8.91
			1522.86		
			1540.80	9.20	9.23
			1550.00		

## Data Availability

The data presented in this study are available on request from the authors.

## Abbreviations

The following abbreviations are used in this manuscript:

WGM	Whispering gallery modes
2PP	Two-photon polymerization
RI	Refractive index
RIU	Refractive Index unit
Q-factor	Quality factor
FDTD	Finite difference time domain
SLD	Superluminescent diode
